# A Dynamic Instrumentation Amplifier for Low-Power and Low-Noise Biopotential Acquisition

**DOI:** 10.3390/s16030354

**Published:** 2016-03-09

**Authors:** Jongpal Kim, Hyoungho Ko

**Affiliations:** 1 Samsung Electronics Inc., Suwon 16678, Korea; jongpalk@samsung.com; 2 Department of Electronics, Chungnam National University, Daejeon 34134, Korea

**Keywords:** biopotential, dynamic instrumentation amplifier, power gating, alternating input, chopper stabilization

## Abstract

A low-power and low-noise dynamic instrumentation amplifier (IA) for biopotential acquisition is presented. A dynamic IA that can reduce power consumption with a timely piecewise power-gating method, and noise level with an alternating input and chopper stabilization technique is fabricated with a 0.13-μm CMOS. Using the reconfigurable architecture of the IA, various combinations of the low-noise schemes are investigated. The combination of power gating and chopper stabilization shows a lower noise performance than the combination of power gating and alternating input switching scheme. This dynamic IA achieved a power reduction level of 50% from 10 µA to 5 µA and a noise reduction of 90% from 9.1 µVrms to 0.92 µVrms with the combination of the power gating and chopper stabilization scheme.

## 1. Introduction

Currently, attempts are being made to perform comfortable and continuous health monitoring in daily life through a wearable system [1,2,3,4,5]. Many biosignals, including electrocardiogram, electroencephalogram, electromyogram, body fat, and heart rate are monitored in contemporary commercialized wearable devices [6,7,8]. Such battery-operated wearable systems inherently require low power, and, thereby, an ultra-low-power health monitoring circuit. An instrumentation amplifier (IA) is one of the most important building blocks for biopotential signal acquisition. High signal-to-noise ratio at the amplifier output is required for further processing in subsequent stages. Generally, a low-noise design requires higher power consumption, because the input-referred noise can be lowered by increasing the power consumption.

In biopotential applications, reducing the flicker noise is an important issue because the flicker noise (1/f noise) is dominant in the low-frequency band. The dominant factors of flicker noise are fluctuations in carrier number and mobility due to the traps at the interface of the silicon and gate oxide [9]. Many research studies have reported a reduction in the flicker noise by various techniques, including correlated double sampling [10], chopper stabilization [11,12,13,14], large signal excitation [15,16], and bulk switching scheme [17].

In this paper, we present a dynamic IA scheme to reduce power consumption in an analog readout channel using power gating (PG). In addition, to recover worsened noise level according to the dynamic IA adaptation, chopper-stabilization (CS), and alternating input switching (AIS) techniques are investigated. The IA is designed to be fully reconfigurable, and can be operated in various combinations with power gating, chopper stabilization, and alternating input switching. In this paper, the optimal combination and operating conditions between PG, CS, and AIS are also investigated.

## 2. Circuit Design

### 2.1. Top Level Architecture

**Figure 1:**
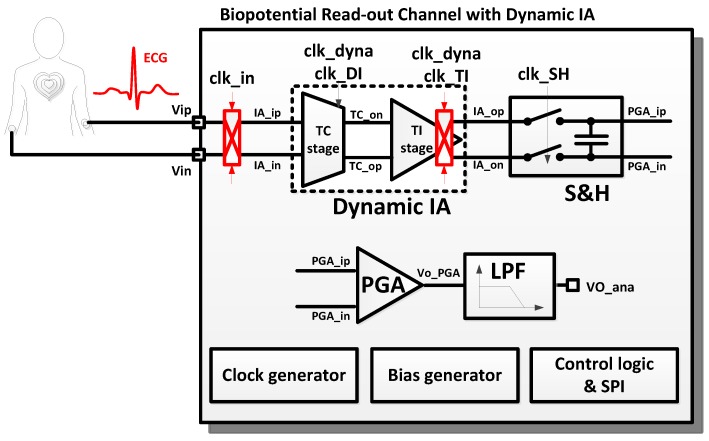
Block diagram of the biopotential readout channel.

**Figure 2:**
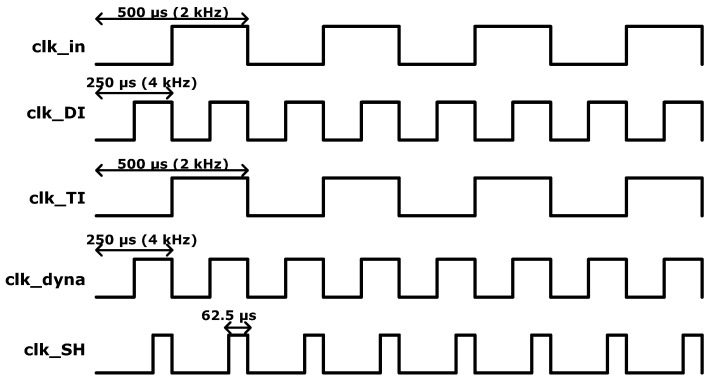
Clock timing examples for the biopotential readout channel.

Figure 1 shows the block diagram of the biopotential readout channel with the dynamic IA. The electrocardiogram (ECG) signal is modulated by the chopping clock, “clk_in”. The chopper operation is controlled by programming the chopping clock. The modulated inputs, “IA_ip” and “IA_in”, are amplified by the dynamic IA. The dynamic IA consists of a transconductance (TC) input stage and transimpedance (TI) output stage. The amplified ECG signal is sampled and held in the “S and H” stage. An additional amplification is performed by the programmable gain amplifier (PGA). Finally, the high-frequency noise in the amplified ECG signal is removed by the low-pass filter (LPF). The readout channel is designed to be fully reconfigurable. The operation mode of each sub-block can be controlled by the control registers. The clock generator can generate the fully programmable clocks using 32-bit bitstream registers. The internal registers can be accessed via the serial peripheral interface (SPI). The clock timing examples for the biopotential readout channel are shown in Figure 2. The biopotential readout channel can be configured in various operating mode for low power and low noise applications. The PG and the appropriate sampling operations are controlled by programming “clk_dyna” and “clk_SH”. The AIS mode is controlled by programming “clk_DI”.

### 2.2. Dynamic IA

**Figure 3:**
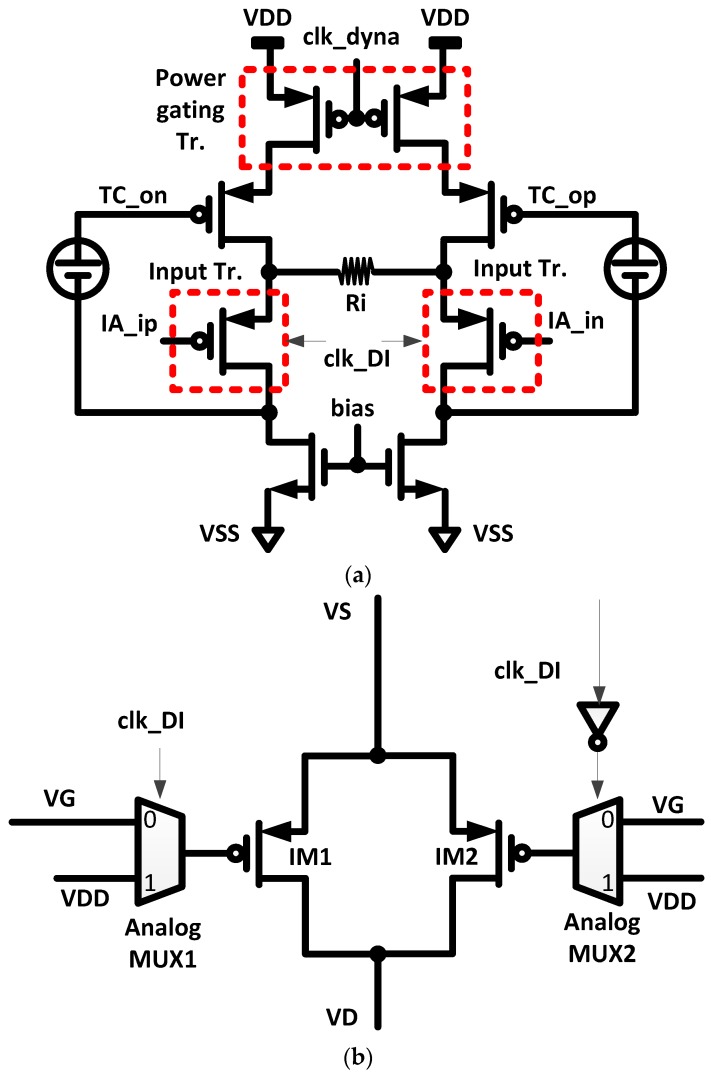
Transconductance (TC) input stage of dynamic IA. (**a**) Schematic of TC input stage; and (**b**) input transistor with alternating input switching (AIS).

The schematic of the TC input stage, which converts the input differential voltage to output differential current, is shown in Figure 3a. The input transistors are operated in a weak inversion region for achieving higher transconductance (g_m_) efficiency. In DC operating points, the *V_GS_* (=*V_GB_*) and *V_TH_* of the input transistors are 255 mV and 329 mV, respectively. The dynamic IA is powered on and off by a control clock signal “clk_dyna”. When the control clock signal “clk_dyna” of a low logic level is supplied to the power gating transistors in the TC stage, the TC stage will be powered off and *vice versa*. According to the duty ratio of the control clock “clk_dyna”, the time-averaged power consumption of the dynamic IA is reduced proportionally. The input stage transistors using alternating input switching (AIS) scheme are composed of two MOS and two analog MUX, as shown in Figure 3b. With the control signal “clk_DI”, either MOS “IM1” or MOS “IM2” is activated. This type of technique with alternating input is known to be helpful in reducing low-frequency band noise [16,18].

**Figure 4:**
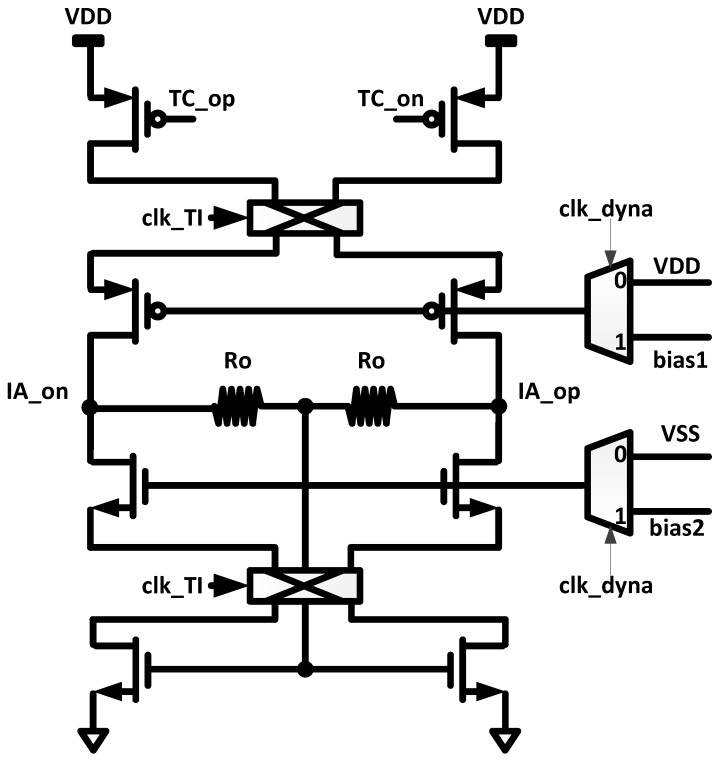
Transimpedance (TI) output stage of dynamic IA.

The schematic of the TI output stage is shown in Figure 4. In this stage, the differential currents from TC stage is converted to output voltages using the resistors, Ro. The resistors, Ro, are also used as resistive common mode feedback. The power gating is also applied in the TI stage. When the power gating clock “clk_dyna” is high, the gate bias voltage is applied, and the TI stage is turned on. When “clk_dyna” is low, the gate bias voltages of PMOS and NMOS become VDD and VSS, respectively; thus, the TI stage is turned off.

The input impedance is mainly affected by input capacitance, input leakage current, and switching frequency of CS or AIS. When the switching frequency of 4 kHz, the simulated input impedance of this circuit in ECG bandwidth is 415 MΩ. The input impedance of 415 MΩ is much larger than the typical impedance of 51 kΩ//47 nF in Ag/AgCl wet electrodes.

## 3. Experimental Results

**Figure 5:**
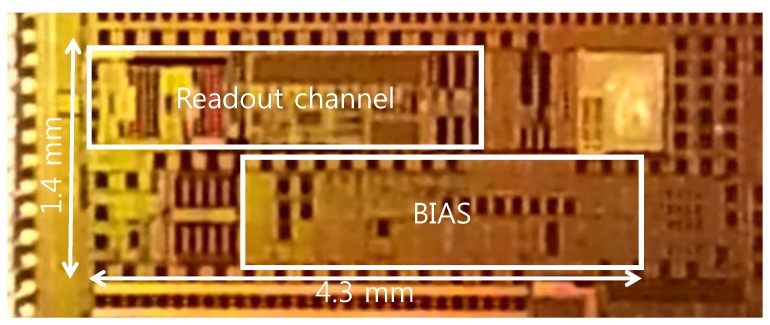
Chip micrograph of the biopotential readout channel with the dynamic IA.

Figure 5 shows the micrograph of the fabricated readout circuit in the 0.13-µm CMOS technology. The chip size is 1.4 mm by 4.3 mm. The supply voltage is 1.2 V, and the supply current without PG is 10 µA.

**Figure 6:**
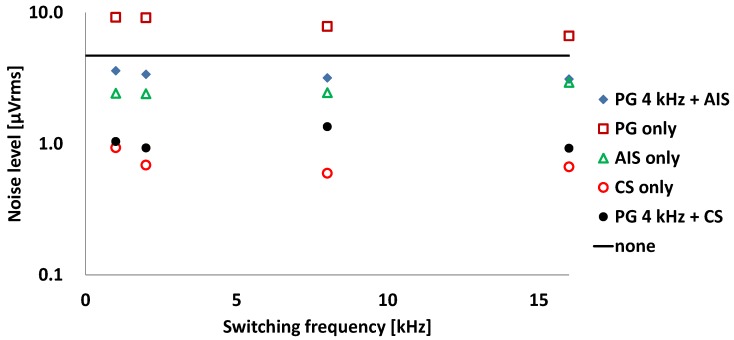
Input-referred noise level according to various operation conditions.

The noise spectrum is measured using a spectrum analyzer, 35670A by Keysight Technologies, Inc. (Santa Rosa, CA, USA), and the input-referred noise is calculated and plotted according to the various operation conditions in Figure 6. The clock configurations for operation conditions are shown in Table 1. The bandwidth of the IA is limited by the fourth-order Bessel low pass filter with 100 Hz cut-off frequency. The bandwidth of the filter can be digitally reconfigurable from 50 Hz to 400 Hz. With the control clocks of “clk_dyna = L”, “clk_LSE = H”, “clk_in = H”, and “clk_TI = H”, the IA is operated in a static condition and the input-referred noise level is 4.7 µVrms marked as “none” in Figure 6. In the static mode with the condition of always “clk_dyna = L”, the conventional chopping technique and the alternating input switching (AIS) technique are helpful in reducing the noise level. To reduce the power consumption, the power gating (PG) technique is applied with a clock signal having a duty ratio of 50% to the control signal “clk_dyna”. The PG technique reduces the power consumption proportionally to the duty ratio of the power gating control signal; however, it increases the noise level by almost double. The noise level of the dynamic IA is investigated with the chopper stabilization (CS) and AIS techniques.

The performance comparisons including noise efficiency factor (NEF) between the various operating conditions at 2 kHz switching frequency are also summarized in Table 2. The NEF is calculated as Equation (1):

(1) $$NEF=V_{ni,rms}\sqrt{\frac{2I_{tot}}{\pi U_{T}\cdot 4kT\cdot BW}}$$

where *V_ni,rms_* is the input referred noise, *BW* is the 3-dB bandwidth of the amplifier, *U_T_* refers to the thermal voltage, and *I_tot_* is the supply current of the amplifier.

The lowest noise level of 0.69 µVrms is measured at the chopper frequency of 2 kHz, wherein the input-referred noise is measured to be 9.1 µVrms with PG only. At the same chopper frequency of 2 kHz, the input-referred noise is reduced to be 3.4 µVrms with the combination of PG and AIS.

The most helpful technique in the dynamic IA is the combination of the PG and CS method. The input referred noise and NEF with the combination of PG 4 kHz and CS 2 kHz are 0.92 µVrms and 7.9, respectively. In terms of the power consumption, PG with the half duty cycle is equivalent to reducing the bias current to one half. When the bias current is reduced to one half, the thermal noise component, which is dominant in chopper stabilized amplifier, will increase approximately by a factor of √2. In this case, the input referred noise with CS with half bias current is expected to be 0.97 µVrms (=√2 ∙ 0.69 µVrms). The combination of PG and CS shows better NEF and lower input referred noise level than the expected values of CS and half bias current.

**Figure 7:**
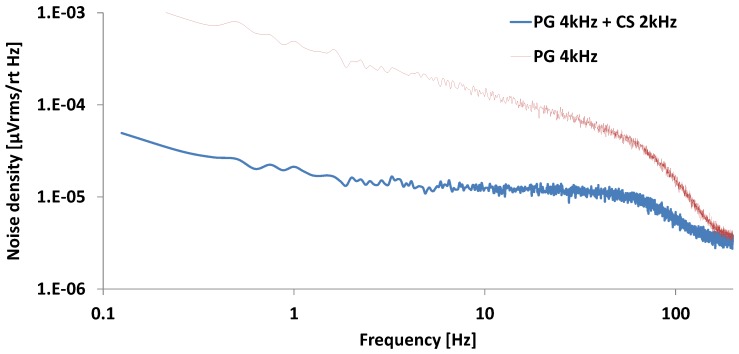
Input-referred noise spectrum of PG 4 kHz and PG 4 kHz + CS 2 kHz.

Figure 7 shows noise spectrum examples in cases of power gating with a 4 kHz clock and a combination of power gating with 4 kHz and chopping with 2 kHz. By adding the chopping technique, the low-frequency band noise in the power gating technique is reduced. The input-referred noise is reduced to 0.92 µVrms with the combination of PG and CS. By applying the half duty-cycled PG and CS, a power reduction of 50% from 10 µA to 5 µA, and a noise reduction of 90% from 9.1 µVrms to 0.92 µVrms can be achieved.

**Figure 8:**
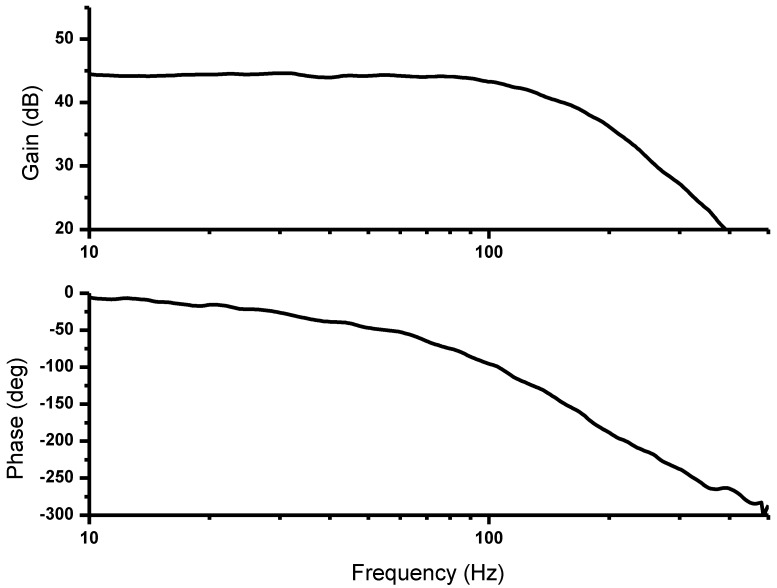
Measured frequency response.

The measured frequency responses including gain and phase plot are shown in Figure 8. The phase distortions are important parameter for high quality ECG recording [19,20]. Although the post-processed digital filter can be used for reducing the phase distortions, the analog filters with inherent phase distortions are used in this system to achieve low power, small size, and real-time signal acquisition, which are important in wearable devices.

**Figure 9:**
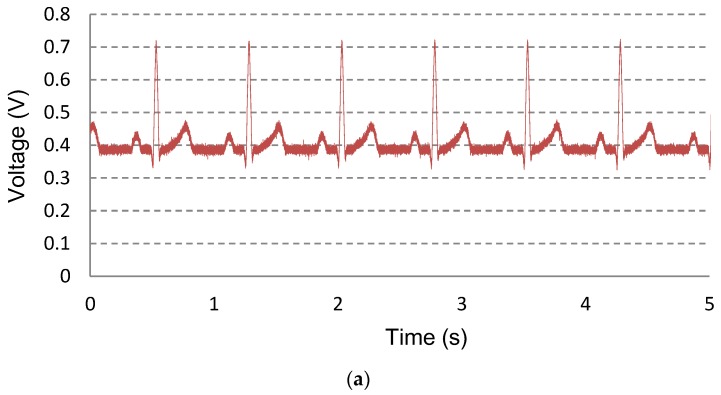

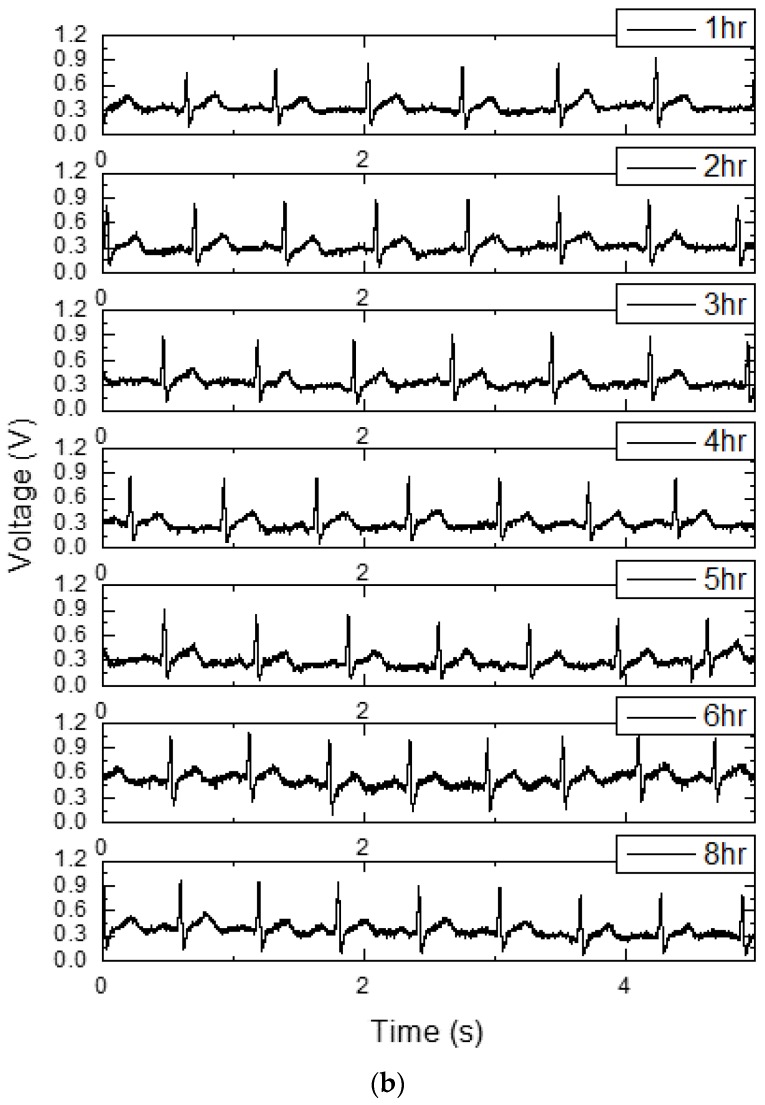
Measured ECG signals in the time domain. (**a**) Measured waveform using ECG simulator; and (**b**) measured ECG signals for eight hours with wet electrodes.

In the time domain, the measured waveform using ECG simulator is shown in Figure 9a. The measured ECG waveforms for 8 h with wet electrodes are shown in Figure 9b. The Ag/AgCl wet electrodes of 3M™ Red Dot™ [21] are used for ECG signal acquisition. The gain of the readout channel is set to 171 V/V. The input ECG signal is preserved with a 50% power reduction compared to the static condition and 90% noise reduction compared to the power gating condition. The transient switching noises due to the switching operations in PG and CS are removed by the fourth-order 100 Hz low pass filter. For example, the 2 kHz switching noises are attenuated by −104 dB (=−80 dB/dec ∙ log(2 kHz/100 Hz) dec). The bandwidth of the filter can be digitally reconfigurable from 50 Hz to 400 Hz.

**Figure 10:**
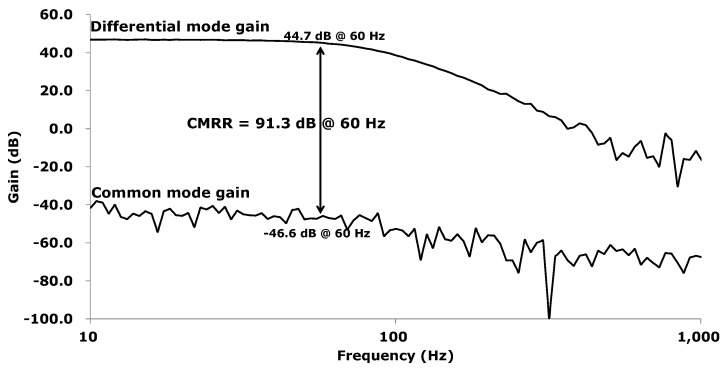
Measured CMRR.

The measured common mode rejection ratio (CMRR) is shown in Figure 10. At 60 Hz, the differential mode gain and the common mode gain are measured to be 44.7 dB and −46.6 dB; thus, the CMRR is calculated to 91.3 dB.

## 4. Conclusions

A low-noise and low-power dynamic IA scheme was presented. A dynamic IA that can reduce power consumption with a timely piecewise power-gating method and noise level with an alternating input and chopper stabilization technique is fabricated with a 0.13-μm CMOS. The combination of power gating and chopper stabilization results in a lower noise performance than the combination of power gating and alternating input switching scheme. With the combination of power gating and chopper stabilization, the supply current is reduced from 10 µA to 5 µA, and the input-referred noise is reduced from 9.1 µVrms to 0.92 µVrms. The power consumption and noise level of the fabricated chip are summarized and compared with the recently-published results summarized in Table 3. In our proposed architecture, we have shown that the dynamic IA technique can achieve a 50% reduction in power consumption and recover the signal-quality deterioration by 90%.

## Figures and Tables

**Table 1:** Clock configurations for various operation conditions.

	PG 4 kHz + AIS	PG only	AIS only	CS only	PG 4 kHz + CS
clk_in	Static “H”	Static “H”	Static “H”	Chopper freq. clock	Chopper freq. clock
clk_DI	AIS freq. clock	Static “H”	AIS freq. clock	Static “H”	Static “H”
clk_TI	Static “H”	Static “H”	Static “H”	Chopper freq. clock	Chopper freq. clock
clk_dyna	4 kHz clock	PG freq. clock	Static “H”	Static “H”	Static “H”
clk_SH	4 kHz clock with 1/4 duty ratio	PG freq. clock with 1/4 duty ratio	Static “H”	Static “H”	4 kHz clock with 1/4 duty ratio

**Table 2:** Comparison of operation conditions at 2 kHz switching frequency.

	PG 4 kHz + AIS 2 kHz	PG only 2 kHz	AIS only 2 kHz	CS only 2 kHz	PG 4 kHz + CS 2 kHz
Input noise (μVrms)	3.4	9.1	2.4	0.69	0.92
Supply current (μA)	5	5	10	10	5
Bandwidth (Hz)	100	100	100	100	100
NEF	29.8	79.9	29.8	8.6	7.9

**Table 3:** IA performance summary and comparison with previous works.

	This Work	[22]	[23]	[24]
Technology	130 nm	180 nm	65 nm	180 nm
Supply	1.2 V	1.2 V	1 V	1 V
IA current	10 µA (static mode) 5 µA (half duty-cycled power gating mode)	5 µA	1.8 µA	3.5 µA
Input-referred noise (~100 Hz)	0.6 µVrms (static mode) 0.9 µVrms (half duty-cycled power gating mode)	1.3 µVrms	6.7 µVrms	1.3 µVrms
Input impedance	415 MΩ (simulated)	1 GΩ	N/A	700 MΩ
CMRR	91.3 dB	120 dB	134 dB	60 dB
